# Physical Education in Elementary Schools

**Published:** 1904-04-30

**Authors:** 


### Physical Education in Elementary Schools.

The report of the committee appointed last year to inquire into the suitability of the Board of Education's model course of physical training, which has just been issued, contains a great deal that is highly suggestive to all who have a regard for the national standard of physique and to medical men in particular. The committee, as a result of their investigations, come to the conclusion that the Board of Education's model course is not suitable for use in schools, and they further consider that it cannot be modified so as to be rendered acceptable, and should therefore be discarded. Their reasons for this opinion are that the course is not based on a consideration of the function of physical exercise as a necessary element of the course of general education in children. In place of the model course they suggest one founded on rational principles, and aiming at the improvement of the physique of the children. The course recommended contains 109 different exercises, which are arranged to provide for the exercising of all parts of the body. Without entering into details it will be sufficient to observe that each one of them has been devised with a special object in view. Thus there are exercises to counteract the tendency to flat-foot, and to rectify the stooping position acquired by working at desks, while great stress is laid on the value of breathing exercises. Mention is made of the evil results of habitual mouth-breathing, and the committee recommend that the breathing exercises may be begun on the child's first admission to school, the formation of habits of correct nasal-breathing being, as they point out, of as great importance as any other department of infant school work. In addition to drawing up the scheme of exercises the committee have duly considered two other important factors in the physical development of school-children, namely, school and personal hygiene. It is sufficiently obvious that certain children ought at times to be excluded from participation in the exercises; but the committee go a step farther than this and suggest that every case of insufficient feeding or constitutional weakness ought to be reported without delay to the local authority in order that steps may be taken at once to investigate the circumstances and apply a remedy, if a remedy be possible. It is pointed out that the first selection of these defective children must be made by the teachers themselves, and while experienced teachers will readily be able to make these first selections, young teachers may not find the matter so easy. It is therefore proposed that the latter should undergo a special training for this work. Indeed the committee expresses the opinion that suitable instruction in the laws of health and in the outward signs of physical and mental weakness should receive a much more prominent place in the general scheme for the training of teachers than appears to have been the case hitherto. For this purpose, they consider, no mere bookwork instruction such as may be necessary for passing written examinations in physiology and hygiene is sufficient. The instruction should include a certain amount of, so to speak, clinical experience. Students should be taught how to recognise probable deviations from the normal standard of health and physique. They should be able to perceive such signs of defective nutrition and such defects of sight, hearing, and breathing as require medical attention. They should also be familiar with the signs of physical or mental fatigue. It is obvious that such a training as is here indicated cannot, and is not intended to, do more than enable the teacher to select those cases which apparently require further investigation. The committee express the view that further investigation must be made by fully-trained medical men who have made a speciality of this kind of work, and that no form of educational organisation can be considered to be complete which does not make provision for the systematic reference of questions of school hygiene and the special treatment of individual scholars to medical experts. Most thoughtful people will, we think, readily endorse the recommendations made by the committee, the members of which are to be congratulated on the excellent outcome of their labours.

